# Imaging-based evaluation of cervical muscle mass and 6-month survival in males with hypopharyngeal carcinoma

**DOI:** 10.2340/1651-226X.2024.40481

**Published:** 2024-12-17

**Authors:** Jouni Laurén, Harri Keski-Säntti, Antti Mäkitie, Otso Arponen

**Affiliations:** a Department of Otorhinolaryngology-Head and Neck Surgery, University of Helsinki and Helsinki University Hospital, Helsinki, Finland; b Department of Radiology, Faculty of Medicine, University of Helsinki, Helsinki, Finland; c Research Program in Systems Oncology, Faculty of Medicine, University of Helsinki, Helsinki, Finland; d Division of Ear, Nose and Throat Diseases, Department of Clinical Sciences, Intervention and Technology, Karolinska Institute and Karolinska University Hospital, Stockholm, Sweden; e Department of Radiology, Tampere University Hospital, Tampere, Finland; f Faculty of Medicine and Health Technology, Tampere University, Tampere, Finland

**Keywords:** Hypopharyngeal carcinoma, body composition, computed tomography, muscle mass, sarcopenia, cachexia, frailty, malnutrition

## Abstract

**Background:**

A significant proportion of newly diagnosed patients with hypopharyngeal carcinoma (HC) are at risk of early death and may not benefit from cancer treatments. Our objective was to assess whether an image-based evaluation of muscle mass could identify patients at risk of impaired survival.

**Materials and methods:**

This retrospective study consisted of male patients diagnosed with HC treated at Helsinki University Hospital between 2005 and 2014 (*N* = 66). Cross-sectional areas of skeletal muscles at the level of the third cervical vertebra (C3) and at the level of the thoracic aortic apex were analyzed using magnetic resonance images and/or computed tomography images. Survival-based cutoff values for low muscle index values were determined using the receiver operating characteristics curves. Kaplan-Meier analyses and Cox proportional hazard models were used to evaluate the associations between the muscle indexes and survival rates.

**Results:**

Several muscle indexes were associated with 6-month and 5-year survival. The 6-month survival rate of males with a low sternocleidomastoid muscle index (cutoff 1.73 cm^2^/m^2^) was 66%, as opposed to the 97% survival rate for those with an above-the-cutoff muscle index (hazard ratio 13.0 [95% CI 1.5, 116.6]). In a multivariate Cox model adjusted for age, sex, tumor stage, and grade, lower sternocleidomastoid muscle index was significantly associated with decreased 6-month survival.

**Interpretation:**

C3-level muscle indexes, particularly the sternocleidomastoid muscle index, are a promising marker in the identification of patients at risk of early mortality and could add confidence in decision-making when choosing between active and palliative care.

## Introduction

Hypopharyngeal carcinoma (HC) represented 9.1% of all head and neck cancers (HNCs) in 2018 [1]. Despite the treatment of choice transitioning in the 1990s from surgery and postoperative radiotherapy (RT) to larynx-preserving chemoradiotherapy (CRT), the dismal prognosis has improved only slightly [2]. Indeed, the reported mean 5-year survival rates range between 25.7 and 41.3% [3–5].

Malnutrition and weight loss commonly affect patients with cancer [6] and even more typically patients with HC, who are generally older, smokers, and heavy drinkers and who often have significant comorbidities and advanced stage of disease at the time of diagnosis [2]. Malnutrition and malignancies predispose patients to cachexia, a metabolic syndrome where systemic inflammation and involuntary weight loss are key factors [7]. Cachexia is common in HNC patients, with a prevalence of 42% in newly diagnosed patients [8]. Sarcopenia or the depletion of strength, skeletal muscle mass (SMM), and function, is a closely related condition. Cachexia and sarcopenia have been associated with increased mortality, increased complication rate, longer hospital stay, functional impairment, and a decreased quality of life in several studies on patients with cancers [9–11]. Body mass index (BMI) alone cannot be used to assess the loss of muscle mass as patients who lose muscle mass may simultaneously lose adipose tissue or be overweight [12]. Analysis of muscle mass could provide better prognostic value of the outcomes of patients. Indeed, SMM depletion has been shown to be an independent predictor of survival among HNC patients [13].

Diagnostic computed tomography (CT) and magnetic resonance images (MRIs) can be used to ‘opportunistically’ evaluate body composition and are regarded as the gold standards for estimating muscle and fat mass [14]. Analysis of cross-sectional area (CSA) of muscles on a single slice at the level of the third lumbar vertebra (L3) is the most widely used method, with a well-established correlation between total body SMM [15]. Additionally, the level of the third cervical vertebra (C3) and different thoracic levels have also been studied. CSA of the muscles at the level of C3 have been demonstrated to strongly correlate with L3-level CSA [16], suggesting its potential in patients with HNC who do not routinely undergo abdominal imaging. CT and MRI can be used interchangeably to determine C3-level muscle mass from imaging examinations [17]. Moreover, adipose tissue can also be quantified. Both decreased C3-level SMM and decreased chest level subcutaneous adipose tissue have been associated with impaired survival in HNC patients [18].

Our objective was to assess whether body composition measurements performed at C3-level and chest-level could help identify patients with HC who have impaired survival. We hypothesize that the identification of patients with short survival could help to individualize the treatment and target supportive care for patients at risk of adverse outcomes.

## Materials and methods

### Study design and patients

This retrospective longitudinal cohort study enrolled all patients with a new, biopsy-proven HC treated at the Helsinki University Hospital (HUS) between 2005 and 2014. The referral area covers approximately 2.2 million people. The study was approved by the HUS institutional review board (study number: HUS 126/2021), and the need for written informed consent for this retrospective study was waived in accordance with national laws and regulations. The data on patient and tumor characteristics were collected from hospital records and images acquired from the hospital’s Picture Archiving and Communications System (PACS).

We included all patients with HC who had either neck area CT or MRI scans as part of the diagnostic workup (i.e., before treatment initiation). Most of the patients also had a pre-treatment chest CT scan that was used to study the role of thoracic body composition measurements in HC. Neck MRI scans without T1-weighed axial (T1W) MRI images and chest CT scans acquired with arms-down positioning were excluded. Finally, as height is needed for the normalization of muscle and adipose tissue mass, patients with missing height data were excluded. The patient selection is presented in Supplementary Figure 1.

### Clinical parameters

Collected clinical data included age at diagnosis, sex, weight, height, smoking status, tumor grade, and overall stage of the disease. HCs were staged according to the 7th edition of the Union for International Cancer Control (UICC) TNM staging system. We retrieved the information on performance status according to the Eastern Cooperative Oncology Group (ECOG) performance status scale, the goal of treatment (curative or palliative), and the given oncologic treatment (none vs. RT or CRT). Multi-class variables were dichotomized into two groups due to the limited number of test subjects: T-stages were grouped into categories consisting of T1–2 and T3–4 tumors, overall stages were grouped into categories consisting of stage 1–2 and 3–4 diseases, grades were grouped into categories consisting of grade 1–2 and 3–4 diseases, and the ECOG performance status was divided into categories consisting of performance status 0–1 and 2–4.

The patients were followed for at least 8 years or until death, whichever occurred first. We defined three different survival timepoints for the analyses: 6-month overall survival (OS), 5-year OS, and OS. Six-month mortality is a commonly used parameter in head and neck oncology to indicate patients who might have been offered alternative management options including palliative care instead of heavy treatment interventions with curative intent [19, 20].

### Clinical CT and magnetic resonance imaging protocols

In this retrospective longitudinal cohort, patients were imaged with different scanners and modalities. All cervical areas were scanned with neck tumor protocols. Cervical CT images were of diagnostic quality. Field strengths and imaging parameters of MRI scans differed but included high-resolution T1W axial images without fat saturation per inclusion criteria. Slice thicknesses in the cervical area ranged between 3 and 5 millimeters.

### Image analysis

One observer analyzed the body composition metrics with 3DSlicer [version 4.11.20210226, https://www.slicer.org/]. In CT images, areas of interest were delineated manually on single slices using the Hounsfield Unit (HU) threshold (–29 to +150 for muscle tissue and –190 to –30 for adipose tissue, respectively) [21]. CT images were included in the study whether iodine-based contrast media was used or not. The same threshold limits were used irrespective of the use of contrast agent. The reader manually corrected the segmentations after the threshold-based semiautomatic segmentation. As tissue intensity level varies between different MRI scanners, tissue compartments were delineated manually on MRI images.

**Figure 1:**
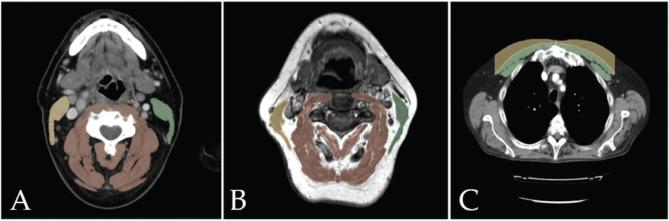
Illustrative examples of the segmented compartments. Images A and B represent the delineated sternocleidomastoid (yellow and green) and PSM (brown) areas of two patients on CT (A) and MRI (B), respectively. The pectoral muscles (green) and thoracic subcutaneous adipose tissue (yellow) compartments are presented in image C. MRI: magnetic resonance images.

Cervical muscles were delineated on a single CT or MRI slice at C3-level. The slice was selected using the method described by Swartz et al.: image stacks were scrolled from the caudal to the cranial direction until the entire vertebral arch and both transverse processes of the C3 vertebra were visible [22]. If the structures were suboptimally visible due to, for example, oblique axial plane compared to the vertebrae or thick slices (5 mm), the observer chose the slice that best fulfilled the criteria. The measured neck muscle compartments were analyzed using the following groups: the bilateral sternocleidomastoid muscles (SCMs), paraspinal muscles (PSMs), and the total C3-level skeletal muscles as a sum of the SCM and PSM areas (Figure 1). Delineated PSMs included the interspinalis cervicis, levator scapulae, longissimus capitis, longissimus cervicis, longus capitis, longus colli, multifidus, rotatores cervicis, semispinalis capitis, semispinalis cervicis, spinalis capitis, spinalis cervicis, splenius capitis, splenius cervicis, and trapezius muscles. We performed the analyses separately for each of the muscle groups (SCMs alone, PSMs alone, and the total C3-level SMM) to evaluate them in prognostication as it is not unequivocal what muscle groups to use in the analyses. If the tumor infiltrated one of the SCM muscles radiologically, we used the area of the contralateral SCM multiplied by two.

Thoracic CT scans were analyzed for the pectoral muscle (PM) and subcutaneous adipose tissue areas. Slice selection was performed as described by Diaz et al.: the stacks were scrolled in the cranial direction and the first slice above the apex of the aortic arch was chosen for analysis [23]. The PM area was measured as the sum of the bilateral pectoralis major and minor muscle areas. The subcutaneous adipose tissue area was measured on the same slice as the area between the skin and the PM; the most lateral aspects of the PM were used as borders, as described by Pishgar et al. [24] (Figure 1).

### Muscle and fat indexes

All segmented muscle or adipose tissue areal values were normalized for stature by dividing the measured areas (cm^2^) by the patients’ squared height (m^2^). The MRI and CT indexes were analyzed separately. Additionally, as we assumed that CT and MRI metrics are interchangeable for body composition analysis, a third group of variables was introduced for cervical compartments, namely ‘MRI-preferred variables’, which consist of a combination of methods. MRI-based values were primarily used, and if there were no MRI images, values were measured from CT images.

### Statistical analysis

Continuous variables are presented as means and standard deviations (SDs) unless otherwise specified. Categorical variables are presented as absolute values and percentages.

To evaluate the associations between radiological indexes and clinical parameters, the equality of variances between groups was first assessed using Levene’s test. If the variances were found to be equal, the independent-sample *T*-test was utilized to analyze differences between two groups. If equal variances were not assumed, Welch’s *T*-test was used instead. The independent-sample *T*-test was also utilized to assess differences in radiological indexes between the sexes.

Cox analyses for survival metrics were first performed in univariate fashion using continuous variables. Subsequently, we used the receiver operating characteristic (ROC) analysis and Youden’s index method to find optimal cutoff values for the prediction of 6-month survival, 5-year survival, and OS when the continuous variables showed statistical significance. Patients were deemed to have low or high index values when the continuous variables were below and above the cutoffs, respectively, and the univariate Cox models were re-analyzed using the categorized variables. Finally, Cox models were performed in multivariate fashion for continuous variables, adjusting for age, stage, and tumor grade. The decision to adjust the models with age was based on the fact that older patients with cancer have lower muscle mass in comparison to younger patients [25]. Stage and grade were used to adjust the multivariable models because of their clinical relevance. Survival analyses were performed for 6-month survival, 5-year survival, and OS. The Kaplan–Meier estimate is provided to visualize the difference between those with low versus normal muscle mass.

All analyses were performed using SPSS (version 27.0.0.1 for Windows, SPSS Inc., Chicago, USA). Two-tailed *p* values ≤ 0.05 were considered statistically significant.

## Results

### Group characteristics

The study groups comprised a total of 77 patients (66 [85.7%] males and 11 [14.3%] females) with 56 neck MRIs, 21 neck CTs, and 67 chest CTs (Supplementary Figure 1). Because of the low number of female patients, we hereafter describe the statistics for male patients only. The mean age was 64.7 ± 9.3 years. Forty patients (60.6%) had a locally advanced disease according to tumor size (T3–4), and 59 (89.4%) had an advanced stage HC (stage III–IV). Only 3 (4.5%) patients had a distant metastasis at the time of diagnosis. Fifty-four (81.8%) patients were treated with curative intent and 55 (83.3%) patients received either RT or CRT. All curatively treated patients, except for one patient who died before the initiation of treatments, received RT/CRT; two patients treated with palliative intention received RT/CRT for life prolongation. None of the patients were treated with first line systemic therapy only. The clinical characteristics of the patients are summarized in Table 1. The mean index values are reported in Table 2.

**Table 1:** Clinical characteristics of the male study patients.

Characteristic	All patients
Patients (N)	66 (85.7)
Age (years)	64.7 ± 9.3
Weight at diagnosis (kg)	74.3 ± 15.3
BMI at diagnosis (kg/m^2^)	23.8 ± 4.2
Smoking	
Never	4 (6.1)
Current or former	62 (93.9)
ECOG	
0-1	42 (63.6)
2-3	14 (21.2)
T stage	
1-2	26 (39.4)
3-4	40 (60.6)
Overall stage	
1-2	6 (9.1)
3-4	59 (89.4)
Grade	
1-2	24 (36.4)
3	35 (53.0)
Treatment intention	
Curative	54 (81.8)
Palliative	12 (18.2)
Oncologic treatment	
RT/CRT	55 (83.3)
None	11 (16.7)
Distant metastasis	
Yes	3 (4.5)
No	63 (95.5)
Survival (months)	52.8 ± 54.7
Survived ≥ 6 months (n)	56 (84.8)
Survived ≥ 5 years (n)	21 (31.8)

BMI: body mass index; ECOG: Eastern Cooperative Oncology Group; RT: radiotherapy; CRT: chemoradiotherapy.

**Table 2:** Radiological muscle and adipose tissue index values (cm^2^/m^2^) in males.

Muscle or fat index	Males		
MRI	*n*	Mean (cm^2^/m^2^)	Standard deviation (cm^2^/m^2^)
Sternocleidomastoid MI	47	2.1	0.6
Paraspinal MI	47	11.0	2.2
Total C3 MI	47	13.1	2.6
MRI-preferred			
Sternocleidomastoid MI	66	2.1	0.6
Paraspinal MI	66	11.2	2.2
Total C3 MI	66	13.2	2.7
CT			
Sternocleidomastoid MI	19	2.0	0.8
Paraspinal MI	19	11.4	2.4
Total C3 MI	19	13.4	2.9
Pectoral MI	57	10.3	3.0
SATI	57	10.6	4.6

MRI: magnetic resonance image; n: Number of patients; MI: Muscle index; SATI: Subcutaneous adipose tissue; CT: computed tomography.

### Associations between radiological body composition metrics and clinical characteristics

Patients with lower T stage (T1 + T2 vs. T3 – T4 [*p* = 0.004]), lower overall stage (1 + 2 vs. 3 + 4 [*p* = 0.006]), curative treatment intention (curative vs. palliative intent [*p* = 0.003]), and those who received oncological treatments (RT/CRT vs. none [*p* < 0.001]) had a higher SCM index. The PSM indexes were statistically higher among patients who received RT/CRT (*p* = 0.011). Significantly higher total C3 muscle indexes were observed among those with curative treatment intention than those with palliative treatment (*p* = 0.003). In thoracic measurements, non-smokers had higher pectoralis muscle indexes (*p* = 0.027), while patients who received RT/CRT had both higher pectoralis muscle indexes (*p* = 0.021) and higher subcutaneous adipose tissue indexes (*p* = 0.037). These results are reported in Table 3.

**Table 3:** Associations between clinical and radiological parameters in male patients. P-values indicate the statistical significance of the difference between groups. P-values ≤ 0.05 (bolded) were considered statistically significant. In the cervical area, MRI-preferred values are shown.

Anatomic area (cm^2^/m^2^) (*n*)	Cervical												Thoracic							
	Sternocleidomastoid MI				Paraspinal MI				Total C3 MI				Pectoralis MI				SATI			
	n	Mean	SD	P	n	Mean	SD	P	n	Mean	SD	P	n	Mean	SD	P	n	Mean	SD	P
T stage																				
1-2	26	2.3	0.6	**0.004**	26	11.5	2.1	0.280	26	13.8	2.5	0.115	22	10.4	2.0	0.845	22	11.6	3.2	0.175
3-4	40	1.9	0.6		40	10.9	2.3		40	12.8	2.8		35	10.3	3.5		35	9.9	5.3	
Overall stage																				
1-2	6	2.7	0.7	**0.006**	6	11.6	2.1	0.602	6	14.3	2.6	0.285	5	10.0	1.3	0.769	5	10.4	2.1	0.929
3-4	59	2.0	0.6		59	11.1	2.2		59	13.1	2.7		51	10.4	3.1		51	10.6	4.9	
Grade																				
1-2	23	1.9	0.7	0.360	23	10.7	2.2	0.153	23	12.6	2.7	0.168	20	9.8	2.6	0.638	20	9.4	3.8	0.191
3	35	2.1	0.6		35	11.5	2.3		35	13.6	2.8		29	10.2	3.0		29	11.2	5.2	
Treatment intention																				
Curative	54	2.2	0.6	**0.003**	54	11.3	2.1	0.194	54	13.5	2.5	0.077	47	10.6	2.9	0.218	47	10.9	4.4	0.307
Palliative	12	1.6	0.7		12	10.4	2.6		12	12.0	3.3		10	9.3	3.2		10	9.2	5.7	
Oncologic treatment																				
RT/CRT	55	2.2	0.6	**< 0.001**	55	11.5	2.2	**0.011**	55	13.6	2.7	**0.003**	49	10.7	3.0	**0.021**	49	11.1	4.6	**0.037**
None	11	1.4	0.4		11	9.6	1.3		11	11.0	1.5		8	8.1	1.6		8	7.4	3.7	
Metastasis																				
Yes	3	2.5	1.2	0.221	3	14.9	3.3	**0.002**	3	17.4	4.6	**0.005**	3	13.0	4.4	0.118	3	12.8	8.8	0.402
No	63	2.0	0.6		63	11.0	2.0		63	13.0	2.4		54	10.2	2.9		54	10.5	4.4	
ECOG																				
0-1	43	2.1	0.6	0.470	43	11.4	2.4	0.853	43	13.5	2.9	0.751	37	11.0	3.0	0.240	37	11.4	4.8	0.471
2-3	14	2.3	0.5		14	11.5	1.6		14	13.8	1.8		12	9.8	2.9		12	10.3	3.8	
Smoking																				
Current or former	62	2.0	0.6	0.255	62	11.0	2.2	0.143	62	13.1	2.7	0.138	53	10.1	2.9	**0.027**	53	10.4	4.2	0.219
Never	4	2.4	0.2		4	12.7	2.3		4	15.1	2.2		4	13.5	2.4		4	13.4	9.3	

n: Number of patients; MI: Muscle index; SATI: Subcutaneous adipose tissue index; RT: Radiation therapy; CRT: Chemoradiotherapy; ECOG: Eastern Cooperative Oncology Group performance status

### Survival analyses

The mean survival time was 52.8 ± 54.7 months. Ten (15.2%) patients died during the 6-month period, 45 (68.2%) died during the 5-year follow-up period, and 11 (16.7%) patients survived until the end of the follow-up period. Lower MRI- and CT-based and MRI-preferred sternocleidomastoid, and MRI- and MRI-preferred total C3 muscle index variables significantly predicted decreased 6-month survival rates in continuous variable univariate Cox regression analyses. None of the variables predicted significantly decreased 5-year OS. Low CT-based pectoral muscle index significantly predicted decreased OS. Detailed results for continuous Cox models are summarized in Table 4.

**Table 4:** Uni- and multivariate models with continuous variables describing the risk of death in male patients. Statistically significant values (P < 0.05) are bolded. The results are not presented for the indexes if the univariate model was not statistically significant.

		Continuous	
		Univariate HR** (95% CI)	Multivariate* HR** (95% CI)
6-month survival	MRI-Sternocleidomastoid MI	0.13 (0.021, 0.85)	0.12 (0.019, 0.81)
	MRI-Total C3 MI	0.63 (0.40, 1.00)	0.58 (0.32, 1.03)
	MP-Sternocleidomastoid MI	0.11 (0.027, 0.45)	0.088 (0.018, 0.43)
	MP-Total C3 MI	0.70 (0.52, 0.94)	0.70 (0.52, 0.95)
	CT-Sternocleidomastoid MI	0.092 (0.009, 0.93)	0.014 (0.00, 1.17)
Overall survival	CT-Pectoral MI	0.90 (0.82, 0.99)	0.93 (0.83, 1.032)

HR: Hazard ratio; CI: Confidence interval; MP: MRI-preferred; MI: Muscle index

* Multivariate models were adjusted for age, stage, and grade.

** HR represents the hazard ratio for each unit of increase in muscle, fat, or body mass index values.

Although age, stage, and grade were not statistically significant in univariate models, they were included in the multivariate model because they are known to contribute to survival. In the multivariate model, both lower MRI-measured and MRI-preferred sternocleidomastoid index and lower MRI-preferred total C3 muscle index significantly predicted decreased 6-month survival in males (Table 4). None of the variables predicted 5-year survival. For OS, the CT-based pectoral muscle index earlier found to be significant in the univariate model in males was no longer statistically significant in the multivariate model.

**Figure 2:**
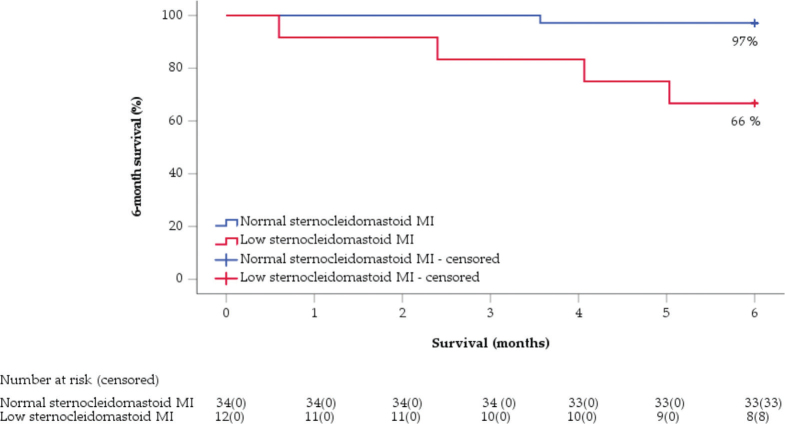
A Kaplan–Meier plot for 6-month survival among males based on a cutoff value for MRI-measured sternocleidomastoid index of 1.73 cm^2^/m^2^. MRI: magnetic resonance images; MI: muscle index.

Uni- and multivariate models for categorical values, along with receiver-operator curves, are presented in Supplementary Table 1 and Supplementary Figures 2 and 3, respectively. Of the categorical muscle indexes, the best predictor of 6-month survival was the MRI-measured SCM index (cutoff 1.73 cm^2^/m^2^), where 6-month survival rates were 97% versus 66% for males with normal vs. low SCM indexes, respectively (Figure 2).

## Discussion

In our study cohort of 66 male patients with hypopharyngeal cancer, both low sternocleidomastoid and total C3 muscle indexes were associated with impaired 6-month OS. The association between lower MRI-measured or MRI-preferred C3-level sternocleidomastoid index, lower MRI-preferred total C3 muscle index and impaired 6-month survival remained statistically significant after adjustment for age and tumor-related factors (stage and grade). Interestingly, BMI was not a statistically significant predictor of survival time. Our results suggest that these indexes are independent prognostic factors for decreased short-interval survival, and the proposed cutoff values are promising in identifying patients at risk of early death. However, given the small patient sample, we suggest caution with the interpretation of particularly the multivariate models. Measuring the muscle mass from diagnostic images, in combination with clinical assessment, may facilitate the identification of patients who are at risk of early death and who may benefit from supportive therapies (e.g., nutritional therapy or physiotherapy) to maintain body weight and muscle mass. It may also add confidence to the decision-making process by stratifying the clinically chosen treatment plan.

Low muscle mass has been confirmed to be a marker of worse prognosis in patients with HNC [7, 26]. Importantly, patients with HNC are at a higher risk of especially severe forms of malnutrition than most other patients with cancer [9]. Patients with HNC frequently suffer from dysphagia because of the tumor growth itself and/or the side effects of the treatments, which often lead to reduced caloric intake, predisposing patients to malnutrition, weight loss, and loss of muscle mass [7, 27].

A significant proportion of patients with HNC and particularly with HC – up to 52.3%, as defined using lumbar imaging-based measurements – may have low muscle mass at the time of diagnosis [28]. Therefore, research on imaging-based body composition analysis in patients with HNC patients remains highly relevant. As patients with HNCs do not routinely undergo abdominal imaging and because there are no well-established cutoff values for cervical muscle indexes, research on imaging-based body composition analysis in patients with HNC has often focused on translating the C3 level measurements to L3 SMM values. These indirectly estimated L3 values have then been used to classify patients into those with low and normal muscle mass according to commonly used cutoff values for low L3 SMM [12, 29]. Although L3 is the most used level for body composition analyses, using indirect measures can lead to over- or under-estimation of muscle mass and may cause the misclassification of some patients with normal muscle mass as having low muscle mass, or vice versa. Indeed, Yoon et al. demonstrated that the correlation between C3 and L3 SMM in patients with low muscle mass was weak (*r* = 0.381) [30]. Vangelov et al. deemed the estimation of L3 SMM from C3 measurements to be unable to replace L3 measurements in their population of 101 patients with HNC [31].

Relatively few publications have focused on direct measures of C3 SMM and their association with survival parameters. Jung et al. assessed directly measured C3 SMM as a prognostic factor of survival in 305 patients with mixed-location HNC with a follow-up of up 98 months; they concluded that low C3 level total muscle mass (cutoff: 56.3 cm^2^) was a significant predictor of decreased OS (HR 5.75 [95% CI 3.40, 9.71]) [32]. Choi et al. assessed cervical skeletal muscle volume and survival in 79 patients with HNC and revealed a significant association between depleted pretreatment cervical muscle volume and survival (HR 3.1 [95% CI 1.2–7.8], but post-treatment SMM and interscan SMM loss were not statistically significant [18]. Widely used cutoff values for low C3 SMM have not been established. A large recent study with 1,415 patients with HNC attempted to standardize cutoff values for total C3 level SMM by defining the cutoff as the mean – 2 SDs (low muscle indexes: males: ≤ 6.8 cm^2^/m^2^ [BMI < 25] and ≤ 8.5 cm^2^/m^2^ [≥ 25 kg/m^2^], females: ≤ 5.3 cm^2^/m^2^ [BMI < 25] and ≤ 6.4 cm^2^/m^2^ [BMI ≥ 25 kg/m^2^]); survival rates were not reported for those with low and normal muscle indexes [33].

Like the muscle indexes determined at C3 level, PM measurements have been correlated to L3 SMM. In two different studies, correlation coefficients of *r* = 0.71 [34] and *r* = 0.45 [35] were found between the pectoralis muscle and L3 muscle indexes, respectively. Compared to the correlation of *r* = 0.79 (in adjusted model *r* = 0.89) reported between C3 and L3 muscle CSA in healthy people [22], the correlation between PM and L3 SMM is weaker, and the authors stated that PM alone should not be used as a substitute for L3 SMM [34, 35]. In our study, low pectoral muscle index significantly predicted decreased OS in univariate analyses. To our knowledge, no research exists on the role of PM measurements in HNC patients.

SMM measurements at the C3 level have demonstrated potential for survival prediction in HNC. In our study, unlike BMI, low SCM and total C3 SMM measurements are associated with impaired 6-month survival rates. Interestingly, muscle mass appeared to be a better prognostic factor for short-term than long-term survival in HC. Further research is warranted to confirm this phenomenon as well as study its likely multifactorial background.

A major limitation of this study is the small number of patients; in particular, the female group consisting of only 11 patients was too small for statistical analyses. The uneven distribution of the sexes is likely due to the higher incidence of HC among males in comparison to females (0.53/100,000 vs. 0.14/100,000 in Finland), whereby 20.9% of new patients are females [3]. In our study, the proportion of female patients was slightly lower (17.3% before the exclusion of unsuitable patients according to the inclusion and exclusion criteria and 14.5% of patients in the final sample). Additionally, a significant amount of missing height data further reduced the number of patients. Heterogenous imaging protocols with both contrast-enhanced and non-contrast CT scans used in combination with MRI scans may affect the reliability of the measurements. Lastly, we only considered pretreatment measurements. SMM assessment could also be used to monitor changes in SMM in consecutive imaging studies. Future research with a larger sample size, particularly with female patients, is warranted to validate the results. Furthermore, the clinical significance of imaging-assessed SMM loss during CRT and the possible increase in imaging-assessed SMM with supportive therapies remain to be studied.

## Conclusions

C3-level muscle indexes, particularly the SCM index, are a promising marker in the identification of patients at risk of early mortality and could add confidence in decision-making when choosing between active and palliative care.

## Supplementary Material

Imaging-based evaluation of cervical muscle mass and 6-month survival in males with hypopharyngeal carcinoma

## Data Availability

The data are not publicly available due to restrictions aiming at ensuring patient confidentiality. Relevant data are presented in the manuscript.

## References

[1] Chow LQM. Head and neck cancer. N Engl J Med. 2020;382(1):60–72. 10.1056/NEJMra1715715 31893516

[2] Keski-Säntti H, Mäkitie AA, Saarilahti K. Intensity-modulated radiotherapy in definitive oncological treatment of hypopharyngeal squamous cell carcinoma. Eur Arch Otorhinolaryngol. 2015;272(9):2489–95. 10.1007/s00405-014-3221-1 25104059

[3] Koskinen AI, Hemminki O, Försti A, Hemminki K. Incidence and survival in oral and pharyngeal cancers in Finland and Sweden through half century. BMC Cancer. 2022;22(1):227. 10.1186/s12885-022-09337-2 35236321 PMC8889707

[4] Kılıç S, Kılıç SS, Hsueh WD, Eloy JA, Baredes S, Woo Park RC, et al. Radiotherapy modality as a predictor of survival in hypopharyngeal cancer. Head Neck. 2018;40(11):2441–8. 10.1002/hed.25360 30306665

[5] Keski-Säntti H, Luukkaa M, Carpén T, Jouppila-Mättö A, Lehtiö K, Mäenpää H, et al. Hypopharyngeal carcinoma in Finland from 2005 to 2014: outcome remains poor after major changes in treatment. Eur Arch Otorhinolaryngol. 2023;280(3):1361–7. 10.1007/s00405-022-07648-5 36094562 PMC9899718

[6] da Fonseca GWP, Farkas J, Dora E, von Haehling S, Lainscak M. Cancer cachexia and related metabolic dysfunction. Int J Mol Sci. 2020;21(7):2321. 10.3390/ijms21072321 32230855 PMC7177950

[7] Mäkitie AA, Alabi RO, Orell H, Youssef O, Almangush A, Homma A, et al. Managing cachexia in head and neck cancer: a systematic scoping review. Adv Ther. 2022;39(4):1502–23. 10.1007/s12325-022-02074-9 35224702 PMC8989808

[8] Jager-Wittenaar H, Dijkstra PU, Dijkstra G, Bijzet J, Langendijk JA, van der Laan BFAM, et al. High prevalence of cachexia in newly diagnosed head and neck cancer patients: an exploratory study. Nutrition. 2017;35:114–18. 10.1016/j.nut.2016.11.008 28241978

[9] Pressoir M, Desné S, Berchery D, Rossignol G, Poiree B, Meslier M, et al. Prevalence, risk factors and clinical implications of malnutrition in French comprehensive cancer centres. Br J Cancer. 2010;102(6):966–71. 10.1038/sj.bjc.6605578 20160725 PMC2844030

[10] Orell-Kotikangas H, Österlund P, Mäkitie O, Saarilahti K, Ravasco P, Schwab U, et al. Cachexia at diagnosis is associated with poor survival in head and neck cancer patients. Acta Otolaryngol. 2017;137(7):778–85. 10.1080/00016489.2016.1277263 28125312

[11] Cruz-Jentoft AJ, Baeyens JP, Bauer JM, Boirie Y, Cederholm T, Landi F, et al. Sarcopenia: European consensus on definition and diagnosis. Age Ageing. 2010;39(4):412–23. 10.1093/ageing/afq034 20392703 PMC2886201

[12] Prado CMM, Lieff JR, Mccargar LJ, Reiman T, Sawyer MB, Martin L, et al. Prevalence and clinical implications of sarcopenic obesity in patients with solid tumours of the respiratory and gastrointestinal tracts: a population-based study. Lancet Oncol. 2008;9(7):629–35. 10.1016/S1470-2045(08)70153-0 18539529

[13] Grossberg AJ, Chamchod S, Fuller CD, Mohamed ASR, Heukelom J, Eichelberger H, et al. Association of body composition with survival and locoregional control of radiotherapy-treated head and neck squamous cell carcinoma. JAMA Oncol. 2016;2(6):782–9. 10.1001/jamaoncol.2015.6339 26891703 PMC5080910

[14] Tolonen A, Pakarinen T, Sassi A, Kyttä J, Cancino W, Rinta-Kiikka I, et al. Methodology, clinical applications, and future directions of body composition analysis using computed tomography (CT) images: a review. Eur J Radiol. 2021;145:109943. 10.1016/j.ejrad.2021.109943 34839215

[15] Shen W, Punyanitya M, Wang Z, Gallagher D, St-Onge MP, Albu J, et al. Total body skeletal muscle and adipose tissue volumes: estimation from a single abdominal cross-sectional image. J Appl Physiol. 2004;97(6):2333–8. 10.1152/japplphysiol.00744.2004 15310748

[16] de Bree R, Meerkerk CDA, Halmos GB, Mäkitie AA, Homma A, Rodrigo JP, et al. Measurement of sarcopenia in head and neck cancer patients and its association with frailty. Front Oncol. 2022;12:884988. 10.3389/fonc.2022.884988 35651790 PMC9150392

[17] Zwart AT, Becker JN, Lamers MJ, Dierckx RAJO, de Bock GH, Halmos GB, et al. Skeletal muscle mass and sarcopenia can be determined with 1.5-T and 3-T neck MRI scans, in the event that no neck CT scan is performed. Eur Radiol. 2021;31(6):4053–62. 10.1007/s00330-020-07440-1 33219847 PMC8128750

[18] Choi Y, Ahn KJ, Jang J, Shin NY, Jung SL, Kim B-S, et al. Prognostic value of computed tomography-based volumetric body composition analysis in patients with head and neck cancer: feasibility study. Head Neck. 2020;42(9):2614–25. 10.1002/hed.26310 32543090

[19] Talani C, Mäkitie A, Beran M, Holmberg E, Laurell G, Farnebo L. Early mortality after diagnosis of cancer of the head and neck – a population-based nationwide study. PLoS One. 2019;14(10):e0223154. 10.1371/journal.pone.0223154 31577831 PMC6774523

[20] Farnebo L, Malila N, Mäkitie A, Laurell G. Early death among head and neck cancer patients. Curr Opin Otolaryngol Head Neck Surg. 2016;24(2):115–20. 10.1097/MOO.0000000000000236 26735585

[21] Peng YC, Wu CH, Tien YW, Lu TP, Wang YH, Chen BB. Preoperative sarcopenia is associated with poor overall survival in pancreatic cancer patients following pancreaticoduodenectomy. Eur Radiol. 2021;31(4):2472–81. 10.1007/s00330-020-07294-7 32974690

[22] Swartz JE, Pothen AJ, Wegner I, Smid EJ, Swart KMA, de Bree R, et al. Feasibility of using head and neck CT imaging to assess skeletal muscle mass in head and neck cancer patients. Oral Oncol. 2016;62:28–33. 10.1016/j.oraloncology.2016.09.006 27865369

[23] Diaz AA, Martinez CH, Harmouche R, Young TP, McDonald ML, Ross JC, et al. Pectoralis muscle area and mortality in smokers without airflow obstruction. Respir Res. 2018;19(1):62. 10.1186/s12931-018-0771-6 29636050 PMC5894181

[24] Pishgar F, Shabani M, Silva TQAC, Bluemke DA, Budoff M, Barr RG, et al. Quantitative analysis of adipose depots by using chest CT and associations with all-cause mortality in chronic obstructive pulmonary disease: longitudinal analysis from MESArthritis ancillary study. Radiology. 2021;299(3):703–11. 10.1148/radiol.2021203959 33825508 PMC8165946

[25] Tolonen A, Kerminen H, Lehtomäki K, Huhtala H, Bärlund M, Österlund P, et al. Association between computed tomography-determined loss of muscle mass and impaired three-month survival in frail older adults with cancer. Cancers (Basel). 2023;15(13):3398. 10.3390/cancers15133398 37444508 PMC10340736

[26] Findlay M, White K, Stapleton N, Bauer J. Is sarcopenia a predictor of prognosis for patients undergoing radiotherapy for head and neck cancer? A meta-analysis. Clin Nutr. 2021;40(4):1711–18. 10.1016/j.clnu.2020.09.017 32994071

[27] de Bree R, van Beers MA, Schaeffers AWMA. Sarcopenia and its impact in head and neck cancer treatment. Curr Opin Otolaryngol Head Neck Surg. 2022;30(2):87–93. 10.1097/MOO.0000000000000792 35255045

[28] Findlay M, White K, Brown C, Bauer JD. Nutritional status and skeletal muscle status in patients with head and neck cancer: impact on outcomes. J Cachexia Sarcopenia Muscle. 2021;12(6):2187–98. 10.1002/jcsm.12829 34676673 PMC8718020

[29] Martin L, Birdsell L, MacDonald N, Reiman T, Clandinin MT, McCargar LJ, et al. Cancer cachexia in the age of obesity: skeletal muscle depletion is a powerful prognostic factor, independent of body mass index. J Clin Oncol. 2013;31(12):1539–47. 10.1200/JCO.2012.45.2722 23530101

[30] Yoon JK, Jang JY, An YS, Lee SJ. Skeletal muscle mass at C3 may not be a strong predictor for skeletal muscle mass at L3 in sarcopenic patients with head and neck cancer. PLoS One. 2021;16(7):e0254844. 10.1371/journal.pone.0254844 34280248 PMC8289025

[31] Vangelov B, Bauer J, Moses D, Smee R. The effectiveness of skeletal muscle evaluation at the third cervical vertebral level for computed tomography‐defined sarcopenia assessment in patients with head and neck cancer. Head Neck. 2022;44(5):1047–56. 10.1002/hed.27000 35138008 PMC9305498

[32] Jung AR, Roh JL, Kim JS, Choi SH, Nam SY, Kim SY. Efficacy of head and neck computed tomography for skeletal muscle mass estimation in patients with head and neck cancer. Oral Oncol. 2019;95:95–9. 10.1016/j.oraloncology.2019.06.009 31345401

[33] Chargi N, Bril SI, Smid EJ, de Jong PA, de Bree R. Cut-off values for low skeletal muscle mass at the level of the third cervical vertebra (C3) in patients with head and neck cancer. Quant Imaging Med Surg. 2022;12(6):3024–33. 10.21037/qims-21-911 35655816 PMC9131345

[34] Sanders KJC, Degens JHRJ, Dingemans AMC, Schols AMWJ. Cross-sectional and longitudinal assessment of muscle from regular chest computed tomography scans: L1 and pectoralis muscle compared to L3 as reference in non-small cell lung cancer. Int J COPD. 2019;14:781–9. 10.2147/COPD.S194003 PMC6452800 31040657

[35] Kim EY, Kim YS, Park I, Ahn HK, Cho EK, Jeong YM, et al. Evaluation of sarcopenia in small-cell lung cancer patients by routine chest CT. Support Care Cancer. 2016;24(11):4721–6. 10.1007/s00520-016-3321-0 27364150

