# Supplementary material for: Imaging-based evaluation of cervical muscle mass and 6-month survival in males with hypopharyngeal carcinoma
Source: Acta Oncol. 2024 Dec 17;63:40481. doi: 10.2340/1651-226X.2024.40481 (PMC11681277; doi:10.2340/1651-226X.2024.40481)
Supplement: Imaging-based evaluation of cervical muscle mass and 6-month survival in males with hypopharyngeal carcinoma [file AO-63-40481-s1.pdf]

Supplementary material has been published as submitted. It has not been copyedited, or typeset by Acta Oncologica

Supplementary materials

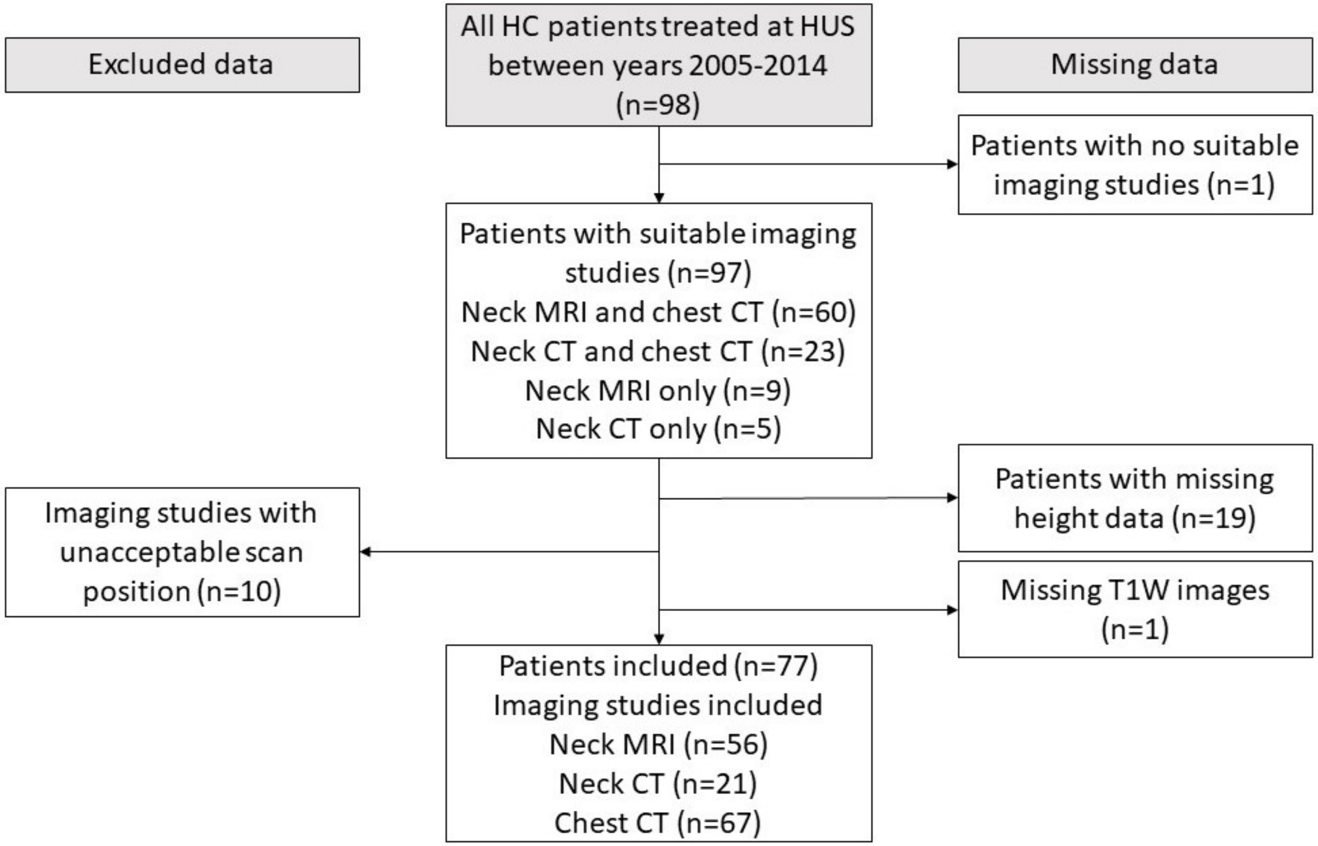

Supplementary figure 1. Patient selection flowchart

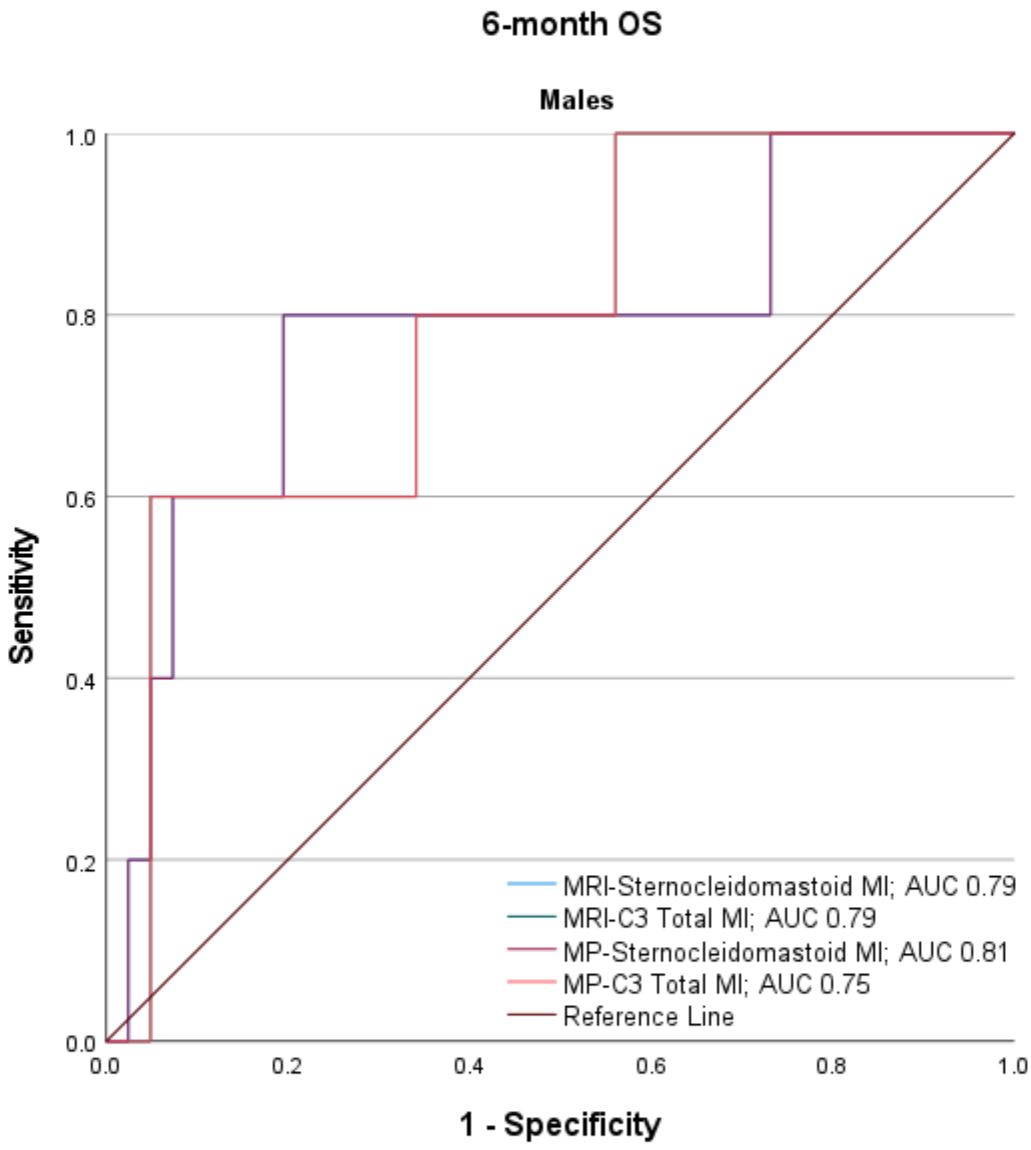

Supplementary Figure 2. Receiver operation characteristic curves for the prediction of 6-month survival in male patients. The analysis was performed for variables that were statistically significant predictors of 6-month OS in continuous variable Cox analysis.

Abbreviations: MP: MRI-preferred; MI: Muscle index; AUC: Area under curve

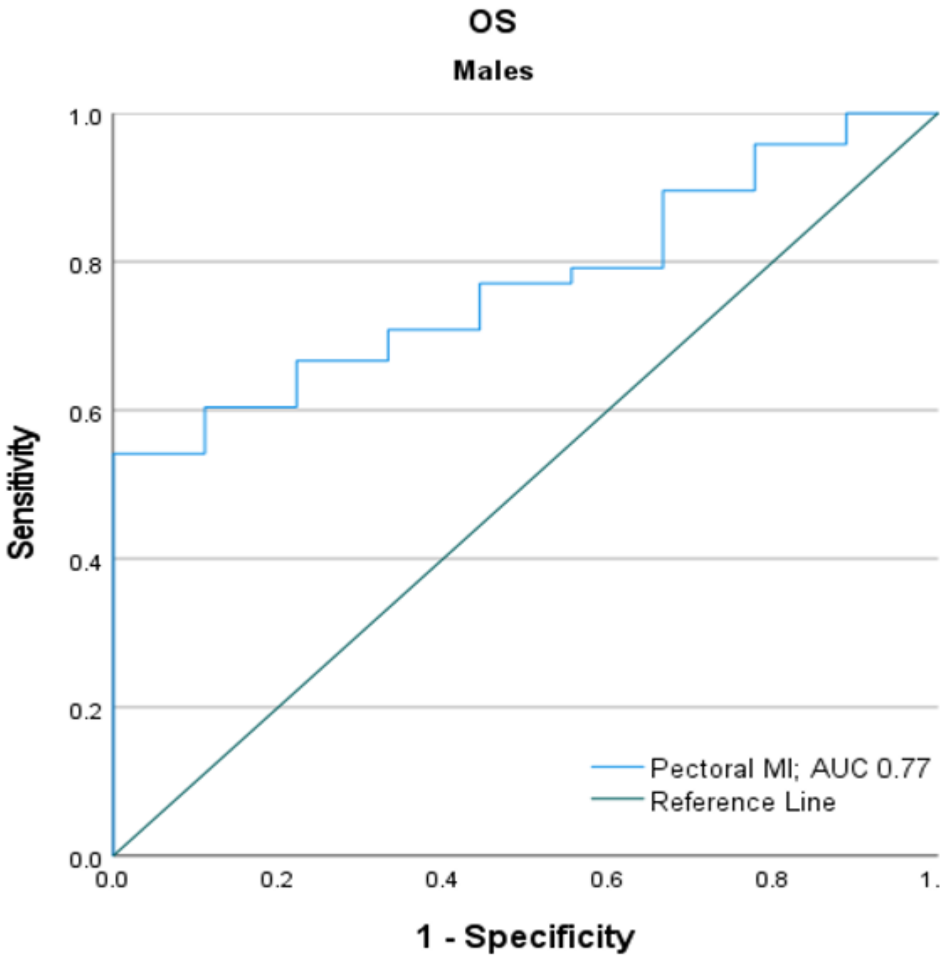

Supplementary Figure 3. Receiver operation characteristic curves for the prediction of overall survival in male patients. All variables that statistically significantly predicted overall survival were included in the ROC analysis.

Abbreviations: MI: Muscle index; AUC: Area under curve

Supplementary Table 1. Uni- and multivariate models with categorical variables describing the risk of death in male patients. Statistically significant values (P < 0.05) are bolded. The results are not presented for the indexes if the univariate model was not statistically significant.

		Categorical				
		Cutoff (cm^2/m^2)	Univariate		Multivariate*	
			Normal muscle mass	Low muscle mass	Normal muscle mass	Low muscle mass
				HR** (95% CI)		HR** (95% CI)
**6-month survival**	MRI-Sternocleidomastoid MI	1.73	REF.	**13.40 (1.50, 120.05)**	REF.	**11.7 (1.29, 106.35)**
	MRI-Total C3 MI	10.16	REF.	**15.60 (2.60, 93.82)**	REF.	**19.9 (2.61, 152.00)**
	MP-Sternocleidomastoid MI	1.73	REF.	**11.49 (2.43, 54.28)**	REF.	**17.44 (2.17, 140.05)**
	MP-Total C3 MI	10.04	REF.	**9.60 (2.74, 33.47)**	REF.	**8.93 (2.36, 33.8)**
	CT-Sternocleidomastoid MI	1.64	REF.	**3.17 (0.42, 23.89)**	REF.	N/C
**Overall survival**	CT-Pectoral MI	9.94	REF.	**2.12 (1.17, 3.82)**	REF.	1.79 (0.94, 3.41)

Abbreviations: HR: Hazard ratio; CI: Confidence interval; MP: MRI-preferred; MI: Muscle index; N/C: Not computable

*Multivariate models were adjusted for age, stage, and grade.

**In categorical variables, HR represents the hazard ratios for "low" muscle index values compared to "normal" index values. Low and normal values were determined with survival-time-specific cutoffs.
